# Editorial: Sustainable nitrogen removal in emerging pollutant contaminated wastewater: technology, application and risk assessment

**DOI:** 10.3389/fmicb.2024.1349185

**Published:** 2024-01-12

**Authors:** Jun Li, Zhaoming Zheng, Yuanyuan Miao, Dingchang Li, Huike Dong, Lijie Zhou, Min Long

**Affiliations:** ^1^National Engineering Laboratory of Urban Sewage Advanced Treatment and Resource Utilization Technology, Beijing University of Technology, Beijing, China; ^2^School of Environmental and Municipal Engineering, Qingdao University of Technology, Qingdao, China; ^3^School of Civil Architecture, East China Jiaotong University, Nanchang, China; ^4^State Key Laboratory of Tibetan Plateau Earth System, Environment and Resources (TPESER), Institute of Tibetan Plateau Research, Chinese Academy of Sciences, Beijing, China; ^5^College of Chemistry and Environmental Engineering, Shenzhen University, Shenzhen, China; ^6^State Key Laboratory of Pollution Control and Resource Reuse, College of Environmental Science and Engineering, Tongji University, Shanghai, China

**Keywords:** nitrogen removal process, sludge granulation process, carbon-neutral, emerging contaminants, microbial population analysis, wastewater treatment

The large-scale release of nitrogen compounds is a major factor for water eutrophication (Li et al., 2023). Traditionally, nitrogen is removed through nitrification and denitrification processes, which leads to external organic carbon addition, high energy consumption, large sludge generation and greenhouse gases emissions (Wu et al., 2023). Many researchers have conducted related research on the autotrophic biological nitrogen removing technology and efficient utilization technology of carbon sources (Al-Hazmi et al., 2023). Anaerobic ammonium oxidation (ANAMMOX) is a crucial autotrophic nitrogen removing process that can reduce the amount of organic matter and energy significantly. Meanwhile, the benefits of aerobic granular sludge (AGS) process are elevated biomass, excellent sludge separation, and small footprint. So far, the comprehensive application of ANAMMOX process and AGS process is facing great challenges (Chen et al., 2022). Besides, the dispersal of emerging organic contaminants in different aquatic ecosystems could have detrimental long-term effects on the global environmental safety (Kumar et al., 2023).

This Research Topic focuses on biological nitrogen removal and emerging contaminants degradation technologies, including ANAMMOX process, AGS process and emerging contaminants removal process. In order to provide readers with an up-to-date understanding of the most fascinating themes, a total of nine articles written by fifty authors were published.

In this topic, Huang et al. systematically reviewed several innovative integrated biological nitrogen removing processes. The performance of short-cut nitrifying and denitrifying, ANAMMOX, simultaneous nitrifying and denitrifying, heterotrophic nitrifying-aerobic denitrifying, AGS, sulfur autotrophic denitrifying, iron autotrophic denitrifying, hydrogen autotrophic denitrifying and bio-electrochemical processes were analyzed in detail. In future, it is worthwhile to take into account the application of cutting-edge bio-denitrifying technology in upgrading urban sewage treatment plants, reducing the greenhouse gases emission and promoting smart water management.

White et al. found that ANAMMOX bacteria could interact with heterotrophic bacteria in a range of competitive and mutualistic ways. Using metagenomic assembly genome analysis, they examined the effects of influent NH_4_^+^-N:NO_2_^−^-N ratios on the microbial population in a laboratory-size ANAMMOX reactor. The NH_4_^+^-N:NO_2_^−^-N ratio was reduced from 1.32 to 1.1, which led to the decrease of ANAMMOX bacterial abundance. Meanwhile, metagenomic sequencing technology revealed that the abundance of bacteria using nrfAH gene related with dissimilatory nitrate reduction to ammonium (DNRA) increased.

Yang et al. evaluated the low concentration of readily biodegradable chemical oxygen demand (COD) on the mainstream ANAMMOX process. In long-term operation, the nitrite oxidation bacteria (NOB) abundance rose to 0.56%. Advanced nitrogen removal was achieved by introducing readily biodegradable organic matter with a COD/nitrogen ratio of 0.9. As a result, the NOB activity was greatly suppressed and high ANAMMOX bacterial abundance (2.48%) was determined.

Aqeel et al. described the settling properties, extracellular polymeric substances components and microbiological community dynamics of activated sludge under the action of organic matter. In autotrophic phase, pin-point granular sludge was obtained in R1 and the SVI_30_ gradually increased to 29 mL/g, indicating the improvement of settling properties. However, the settlement properties and PN:PS ratio of flocs decreased in the heterotrophic phase. Molecular approaches indicated that the pin-point granular sludge's major nitrifying bacteria was *Nitrospira*. Moreover, the predominant ammonium oxidization bacteria in seeding sludge and low ammonium cultivation conditions was *Comammox Nitrospira*.

Xu et al. analyzed the impact of lignocellulose on the AGS's granulation evolution process, structural stability and contaminants removing efficacy. It was shown that lignocellulose served as a skeleton within granules, promoting the development of AGS and improving structural strength, in addition to enhancing the secretion of polysaccharides components in tight extracellular-polymeric materials. Besides, lignocellulose had minimal effect on the removing efficiencies of COD, ${\text{NH}}_{4}^{+}$ -N and ${\text{PO}}_{4}^{3-}$-P, which were more than 95%, 99%, and 92%, respectively. Lignocellulose facilitated the significant proliferation of functional microbes such as *Nitrosomonas, Nitrospira, Candidatus Accumulibacter*, and *Candidatus Competibacter*.

Long et al. revealed the degradation performance of Ag-Bi_3_O_4_Cl plasma photocatalysts for emerging contaminants. The quantity of oxygen-deficient on the surface of catalyst first rose and subsequently dropped as the silver metal level increased. In addition, the light absorption efficiency was increased due to the plasmon resonance effect on the catalyst's surface, which decreased electron-hole pair recombination and increased the migration ability of electron-hole pair. Under optimal Ag-Bi_3_O_4_Cl dose, the removing efficiencies of ciprofloxacin and tetrabromo bisphenol A were 93.8% and 94.9%, respectively.

In the study of Zhang et al., a fungi-algae particle was assembled using *Fusarium* sp. and *Chlorella* sp. to break down polyacrylamide (PAM) and fix inorganic carbon (IC) in synthetic wastewater. It was demonstrated that a mixture of *Fusarium* sp. and *Chlorella* sp. was superior than the single species in terms of PAM degradation and carbon removal. For IC removal performance, the removing rate of the fungal-algal mixture was 38.5% ± 0.08% higher than that of microalgae.

Feng et al. evaluated the impact of synthetic polymers and agricultural wastes on the denitrification performance of seawater circulating treatment. It was found that the carbon releasing ability of agricultural waste was larger than that of synthetic polymers. Corn cobs in agricultural waste is a perfect carbon source for removing nitrogen from seawater aquaculture under low C/N ratio condition.

Wang et al. developed a real-time and unpolluted model for COD detection, which played an important contribution to the early detection of novel organic pollutants. As the organic contaminants and turbidity were commonly absorbed in the ultraviolet wavelength range, the accuracy of detection was inevitably affected. In their study, the superposition principle was used to deduct the turbidity-induced absorbance from the overlapping spectra.

This Research Topic addressed the most recent advancements on the nitrogen removing and emerging contaminants degradation technologies for low C/N ratio sewage treatment. The suitable operating parameters, treatment efficiency and microbial collaboration mechanism were well elucidated through laboratory experiments. It is necessary to further conduct new biological nitrogen removal technology research combined with practical engineering in the future.

## Author contributions

JL: Writing—original draft, Writing—review & editing. ZZ: Writing—original draft, Writing—review & editing. YM: Writing—review & editing. DL: Writing—review & editing. HD: Writing—review & editing. LZ: Writing—review & editing. ML: Writing—review & editing.
